# Identification of a Vibration Regime Favorable for Bone Healing and Muscle in Estrogen-Deficient Rats

**DOI:** 10.1007/s00223-013-9706-x

**Published:** 2013-02-17

**Authors:** Marina Komrakova, Stephan Sehmisch, Mohammad Tezval, Jan Ammon, Peggy Lieberwirth, Cordula Sauerhoff, Lukas Trautmann, Michael Wicke, Christian Dullin, Klaus M. Stuermer, Ewa K. Stuermer

**Affiliations:** 1 Department of Trauma Surgery and Reconstructive Surgery, University Medical Center Goettingen, Robert-Koch 40, 37075 Goettingen, Germany; 2 Department of Animal Science, University of Goettingen, Albrecht-Thaer-Weg 3, 37075 Goettingen, Germany; 3 Department of Radiology, University of Goettingen, Robert-Koch 40, 37075 Goettingen, Germany; 4 Institute for Research in Operative Medicine, Faculty of Health, School of Medicine, Witten/Herdecke University, Cologne, Germany

**Keywords:** Whole-body vibration, Osteoporosis, Bone healing, Muscle, Ovariectomized rat

## Abstract

Numerous whole-body vibration (WBV) devices of various forces are available on the market, although their influence on the musculoskeletal system is not yet understood. The effect of different WBVs on bone healing and muscle function was evaluated in rats ovariectomized at 3 months of age. 2 months after ovariectomy, bilateral metaphyseal tibia osteotomy and T-plate osteosynthesis were performed. Rats were divided into groups: intact, OVX, and OVX exposed to vertical WBVs of 35, 50, 70, or 90 Hz (experiment 1) or horizontal WBVs of 30, 50, 70, or 90 Hz (experiment 2) 5 days after osteotomy (0.5 mm, 15 min/day for 30 days). The tibia and gastrocnemius and soleus muscles were collected. Vertical vibrations (>35 Hz) improved cortical and callus densities, enlarged callus area and width, suppressed the tartrate-resistant acid phosphatase gene, enhanced citrate synthase activity, accelerated osteotomy bridging (35 and 50 Hz), upregulated the osteocalcin (Oc) gene (70 Hz), and increased relative muscle weight (50 Hz). Horizontal vibrations reduced cortical width (<90 Hz) and callus density (30 Hz), enhanced alkaline phosphatase (Alp) gene expression (50 Hz), decreased the size of oxidative fibers (35 and 70 Hz), and increased capillary density (70, 90 Hz). Biomechanical data; serum Oc, Alp, and creatine kinase activities; body weight; and food intake did not change after WBVs. Vertical WBVs of 35 and 50 Hz produced more favorable results than the higher frequencies. Horizontal WBV showed no positive or negative effects. Further studies are needed to elucidate the effects of WBV on different physiological systems, and precautions must be taken when implementing WBV in the treatment of patients.

## Introduction

Whole-body vibration (WBV) treatments are becoming increasingly common in sports and rehabilitation [1]. WBV has been introduced as a noninvasive, nonpharmacological therapy for osteoporosis due to its anabolic effects on musculoskeletal tissue [2]. Numerous clinical studies have shown a significant improvement in bone mineral density (BMD) after WBV treatments in postmenopausal women with osteopenia and osteoporosis [3]. Maintenance of bone strength and density during aging is highly dependent on the maintenance of adequate muscle mass and function. A loss of muscle strength leads to physical frailty, which increases the risk of falls that lead to osteoporotic fracture. Estrogen status has a profound influence on fracture healing and muscle properties. When a fracture occurs in estrogen-deficient individuals, bone healing is delayed and the recovery of the muscle after inactivity is slowed [4, 5].

Physical exercise is considered an effective strategy for the recovery of muscle and bone after trauma, and it is frequently recommended in general practice. However, in elderly people, exercises may induce arduous stress on bone and increase the risk of injuries, especially in osteoporotic patients. Furthermore, studies have shown that it is difficult to encourage elderly people to exercise. Elderly women are often too weak to perform physical exercise. For these patients, WBV may be an option to stimulate the recovery rate of muscle and bone regeneration. Mechanical stimuli have been shown to promote fracture healing by stimulation of new bone formation [6]. Muscle mass, muscle strength, and muscular performance and balance have been enhanced after WBV treatments [7].

Today, many WBV devices of various forces (vertical, sinusoidal, or horizontal) are available on the market; however, it is unknown what type of vibratory signal is necessary to achieve a beneficial response from muscle and bone [8]. The effects of different vibration-loading parameters (frequency, magnitude, duration) on the musculoskeletal system are well understood, partly because of the large variability in the vibration regimes used in clinical and experimental trials. Different vibration regimes can either stimulate bone healing or impair the bone regeneration process [9, 10]. Similarly, WBV may exert beneficial or adverse effects on muscle tissue [10].

We investigated the effect of vibration regimes of different frequencies (30–90 Hz) and types (vertical or horizontal) on bone healing and muscle function in ovariectomy-induced osteopenic rats.

## Materials and Methods

### General Procedures

All procedures were approved by the local regional government according to the German animal-protection laws. Animal experiments and data analyses were performed at the University Medical Center of Goettingen. 3-month-old female Sprague–Dawley rats (Harlan Winkelmann, Borchen, Germany) were housed in groups of four or five in standard cages under a 12-hour dark–light regime at a temperature of 22 ± 1 °C. Animals received a standard pellet diet (ssniff Special Diet; ssniff, Soest, Germany) and water without restrictions throughout the experiments. Food consumption and body weight (BW) were recorded weekly during the experiment. One week after delivery and acclimatization, the rats designated as healthy controls were left intact. Rats that were assigned to the severe osteoporotic groups underwent bilateral ovariectomy under intraperitoneal ketamine and xylazine anesthesia (115 and 8 mg/kg BW, respectively). Rats were housed for 8 weeks to induce osteoporotic conditions in the ovariectomized (OVX) group [11]. Thereafter, a bilateral osteotomy of the tibial metaphysis with T-plate osteosynthesis was performed under ketamine and xylazine anesthesia in all rats [12]. Briefly, both tibiae were osteotomized transversally 7 mm distal of the knee surface with the aid of a pulsed ultrasound saw (Piezosurgery^®^; Mectron Medical Technology, Carasco, Italy). A 5-hole, T-shaped titanium plate fixed the osteotomized bone ends with the aid of four screws at the ventromedial aspect of the tibia. Plates and screws were obtained from Stryker Trauma (Selzach, Switzerland). During the operation, rats received perphenazine (Decentan; Merck, Darmstadt, Germany) and carprofen (Rimadyl; Pfizer, Karlsruhe, Germany) subcutaneously (100 and 4 mg/kg BW, respectively). Carprofen was given twice a day for 2 postoperative days. Fluorescent dyes were injected subcutaneously to label the new bone formation at the site of the osteotomy. Xylenol orange (90 mg/kg BW, Merck) was injected on day 13, calcein (10 mg/kg BW; Chroma/Waldeck, Muenster, Germany) was injected on day 18, alizarin complexone (30 mg/kg BW, Merck) was injected on days 22 and 24, and tetracycline (25 mg/kg BW; Roth, Karlsruhe, Germany) was injected on day 35 after osteotomy [13]. 5 days after osteotomy, rats were exposed to the different vibration treatments.

In experiment 1, the effect of vertical WBV at different frequencies on bone healing and muscle was studied. There were six groups of rats (15 rats each). Group 1 served as an intact control (intact). Group 2 served as an osteoporotic control (OVX). In groups 3, 4, 5, and 6, OVX rats were treated with either 35, 50, 70, or 90 Hz of vertical vibration, respectively. Vibrations were applied using a previously described device [10] consisting of a vibration desk, two alternating current engines, and a force transducer that adjusts the vibration frequency from 35 to 90 Hz.

In experiment 2, the effect of horizontal WBV of different frequencies on bone healing and muscle was investigated. Rats were divided into the following treatment groups (15 rats each): (1) intact rats, (2) OVX, (3) OVX rats treated with 30 Hz horizontal vibration, (4) OVX 50 Hz, (5) OVX 70 Hz, and (6) OVX 90 Hz. Vibration treatments were conducted using a newly developed vibration device (Vibra Maschinenfabrik Schultheis, Offenbach, Germany) consisting of a vibration desk, two alternating current engines attached to the one side of the desk, and a force transducer to change the vibration frequency between 30 and 100 Hz.

In both experiments, rats (seven or eight at a time) were vibrated in a plastic cage (50 × 50 × 25 cm) that was attached to the vibration desk. The vibration regime was as follows: 15 min once a day for 30 days. The amplitude of the vibrations was 0.5 mm. Nonvibrated rats were handled the same as the experimental groups excluding the vibration treatments.

35 days after osteotomy, rats were decapitated. Blood samples were collected and stored at −20 °C until analyses. The uterus was extracted and weighed. Both tibiae were dissected free of soft tissues. The plate and screws were removed. Either the left or right tibia, chosen randomly, was stored at −80 °C for mRNA analyses. The contralateral tibia was stored at −20 °C until micro-computed tomographic (micro-CT), biomechanical, and histological analyses. The left and right muscularis gastrocnemius (MG) and muscularis soleus (MS) were extracted, weighed, and immersed in liquid nitrogen to be further stored at −80 °C for either histological or enzyme analysis.

### Serum Analyses

Analyses of serum alkaline phosphatase (Alp), osteocalcin (Oc) and creatine kinase (Ck) were conducted at the Department of Clinical Chemistry, University Medical Center Goettingen, using an automated chemistry analyzer (Roche/Hitachi Modular) and commercially available kits (Roche) according to the manufacturer’s instructions (Roche Diagnostics, Mannheim, Germany). Alp was measured using a colorimetric assay, Oc was determined using an electrochemiluminescence immunoassay, and Ck was assayed by the “reverse reaction” and activation by acetylcysteine.

### Bone Healing Analyses

Biomechanical testing was performed using a three-point bending test [14] with the aid of a testing machine (type 145660 Z020/TND) and Test Expert Software (Zwick/Roell, Ulm, Germany). The thawed tibia was loaded at the osteotomy line of the ventral metaphysis. Nondestructive measurements were stopped automatically by the software when the elastic deformation reached the end point (yield load). Stiffness was defined as the slope of the linear rise of the curve.

**Fig. 1:**
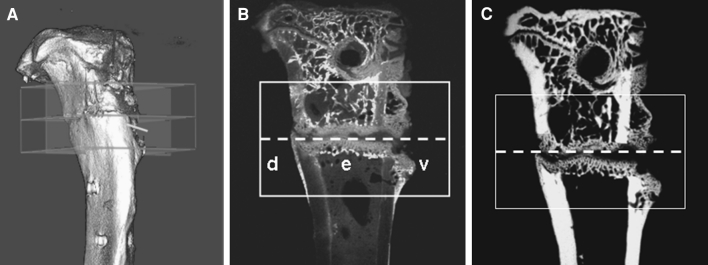
Images of the measurement area extending 2.5 mm proximally and distally (*rectangular frame*) from the osteotomy line (*dashed line*) analyzed with the aid of micro-CT (**a**) and histological analyses of a fluorescence-labeled section (**b**) and its radiograph (**c**). Measurement regions: ventral (*v*), dorsal (*d*), endosteal (*e*) (experiment 2, group 30 Hz)

Thereafter, the tibia was scanned using micro-CT (eXplore Locus SP-Scanner; GE Healthcare, Ontario, Canada) [15]. The scan protocol was as follows: 72 kVp, 90 μA, 1,600 ms exposure time, 360° rotation, 0.029 mm pixel size, and 900 views. A 3D reconstruction was performed with the aid of the MicroView-Program (v2.1.2, GE Healthcare). After reconstruction of the data, the analysis was conducted using a newly developed bone analysis program. The 3D measurement area extended 2.5 mm proximally and distally from the osteotomy line (Fig. 1a). The following parameters were quantified: total bone volume (BV) and mineral density, callus and cortical densities, and bone volume per total volume fraction (BV/TV). The data were converted into BMD (g/cm^3^) using a linear regression equation, BMD = 0.23 × (value + 0.55), that was formulated by measuring three hydroxyapatite standards of several mineral densities.

For histological analyses, the tibia was subjected to sequential ascending concentrations of ethanol and embedded in methylmethacrylate (Merck). Sections of 150 μm thickness were cut longitudinally at a right angle to the implant bed using a diamond saw microtome (SP 1600; Leica Instruments, Nussloch, Germany). The time of the osseous bridging of the osteotomy gap was determined by analyzing fluorochrome-labeled callus tissue using at least 10 sections with the aid of a Leica microscope (Leitz DM RXE) [13]. Three representative central sections of the tibia were microradiographed using the Faxitron Cabinet X-ray system (Hewlett-Packard, Buffalo Grove, IL) and Kodak Industrex film (SR45, 100 NIF; Kodak, Paris, France). Sections and microradiographs were digitalized using a digital camera (Leica DC300F) and a zoom stereo microscope (Leica MZ75) and analyzed using the QWin image analysis program (Leica, Bensheim, Germany). The measurement area extended 2.5 mm proximally and distally from the osteotomy line (Fig. 1b, c). This area was divided into three regions of interest (ROI): (1) ventral, (2) dorsal, and (3) endosteal parts of the tibia (Fig. 1b). The cortical width and density distal to the osteotomy line, periosteal callus width and density, endosteal callus density, and trabecular density and width were measured in microradiographs (Fig. 1c) [10]. The callus area (μm^2^) was measured according to the ROI and fluorescence labeling in histological sections (Fig. 1b). The xylenol orange–labeled area was relatively small and, therefore, assessed along with the calcein-labeled area.

### Analyses of Gene Expression at the Osteotomy Site

The metaphyseal part of the contralateral tibia containing newly formed callus was homogenized using a micro-dismembrator S (Sartorius, Goettingen, Germany). Total cellular RNA was extracted using the RNeasy™ Mini Kit (Qiagen, Hilden, Germany) and reverse-transcribed by Superscript™ RNase H-reverse transcriptase (Promega, Mannheim, Germany). Expression of rat genes Alp, Oc, insulin-like growth factor-1 (Igf-1), receptor activator of nuclear factor kB ligand (Rankl), osteoprotegerin (Opg), and tartrate-resistant acid phosphatase (Trap) was determined using quantitative real-time polymerase chain reaction (PCR) based on SYBR green detection (PCR QuantiTect Sybr^®^ Green Kit, Qiagen) in an iCycler (CFX96, Bio-Rad Laboratories, Munich, Germany). Ready-to-use primer pairs were obtained from Qiagen (QuantiTect Primer Assays). The quantitative real-time PCR was carried out according to the manufacturer’s instructions. Gene expression was calculated using the 2^−ΔΔCT^ method [16], and the results are shown relative to the gene expression in untreated female rats (non-OVX, nonosteotomized) that were of the same age as the experimental animals and of comparable BW. The reference gene was β_2_-microglobulin.

### Muscle Analyses

Muscle samples were cut into serial cross sections of 12 μm thickness using a cryostat (Frigocut 2800E, Leica Instruments).

For analysis of muscle fibers, sections were fixed in a 1 % paraformaldehyde solution (pH 6.6) containing 1 % CaCl_2_ and 6 % sucrose. Thereafter, muscle fibers were stained by incubation in a reduced nicotinamide adenine dinucleotide diaphorase (NADH-diaphorase) solution (pH 7.4), followed by acidic incubation (pH 4.2) and incubation in adenosine-5′-triphosphate solution (pH 9.4) [17]. Muscle fibers were classified as fast-twitch glycolytic (G), fast-twitch oxidative (O), and slow-twitch O types according to Peter et al. [18]. The fast- and slow-twitch O fiber types were combined for analysis. Cross-sectional areas (CSAs) of at least 90 O and 90 G fibers determined within three fields of the cross section (30 fibers/type within a field of 1 mm^2^) were measured. In the MS, only O fibers were measured as the MS mainly consists of these fibers [19].

For analysis of muscle capillaries, sections were fixed in 100 % ethanol/chloroform/glacial acid at a ratio of 16:3:1 at 4 °C for 1 h. Thereafter, sections were incubated in 0.3 % α-amylase solution, treated with 1 % periodic acid, and stained in Schiff’s reagent solution (Roth) [20]. Finally, sections were treated with a 10 % potassium sulfite solution. The capillaries and fibers found in three randomly selected fields (1 mm^2^ each) within a cross section were counted. The ratio of capillaries to muscle fibers was determined in the analyses.

For the analyses of muscle enzymes, muscle samples were weighed and homogenized in ice-cold Chappel-Perry medium (0.1 M KCl, 0.05 M Tris, 0.01 M MgCl_2_ × 6H_2_O, 1 mM EGTA, pH 7.5) using a Potter-S-homogenizer (B. Braun Biotech International, Melsungen, Germany). Enzyme activities were assayed using a photometer (LP6; Hach Lange, Duesseldorf, Germany). Assays were performed in triplicate at 30 °C. Lactate dehydrogenase (LDH) was measured as described previously [21]. Citrate synthase (CS) activity was assayed according to Faloona and Srere [22]. Complex I activity was assayed according to Hatefi and Stiggall [23]. Protein content was determined using a BCA™ Protein Assay Kit (Pierce, Rockford, IL), a multilabel reader (Perkin Elmer Precisely Victor X4), and software version 4.0 (Perkin Elmer Life and Analytical Science, Turku, Finland). The activity of the enzymes was calculated relative to the protein content.

### Statistical Analyses

Statistical analyses were conducted using the SAS program (version 9.1; SAS Institute, Cary, NC). ANOVA (*P* < 0.05) was applied to reveal the impact of the treatments on the respective variables. The effects of treatment duration were taken in the model for analyses of food intake and BW variables. The BW of rats taken as a covariate in the analysis of muscle weight was found to be significant (*P* < 0.05). Therefore, the muscle weight to BW ratio (MG/BW, MS/BW) was analyzed. Differences between individual means were estimated using the Scheffé test (*P* < 0.05). Relative gene expression was analyzed using the nonparametric Kruskal–Wallis test and Dunn’s multiple comparison test (GraphPad Prism 4.0; GraphPad Software, La Jolla, CA).

## Results

### Body Weight, Food Intake, and Uterus Weight

In both experiments, ovariectomy significantly affected the BW of rats, whereas the vibration treatments did not change it. At the beginning of the experiments, the mean BW did not differ (*P* > 0.05) among the groups (238 ± 3 g in experiment 1 and 240 ± 3 g in experiment 2 on average). The BW of OVX rats increased sharply after ovariectomy to 332 ± 5 g after 8 weeks. After osteotomy, BW dropped significantly (309 ± 5 g, week 9) and recovered toward the end of the experiment (320 ± 5 g, week 13). In intact rats, BW also increased during the first 8 weeks (237 ± 4 g, week 0; 276 ± 4 g, week 8 on average), whereas after osteotomy, it did not change significantly over the next 5 weeks.

Vibration treatments had no effect on food intake in either experiment. In OVX rats, food intake was significantly higher than that in intact rats (OVX 23 ± 0.5, intact 18 ± 0.4 g/rat daily on average, respectively). After osteotomy, no differences were revealed among the groups; food intake dropped to 16 ± 0.7 g/rat daily (week 9). During the next 4 weeks, food intake increased to the level observed at the time of osteotomy (21 ± 2 g/rat daily, week 13).

The weight of the uterus was significantly higher in intact rats than in OVX rats, and vibration had no effect (experiment 1, intact 604 ± 50 mg, all OVX groups 105 ± 4 mg; experiment 2, intact 550 ± 35 mg, all OVX groups 119 ± 5 mg).

### Serum Analyses

Vibration treatments had no effect on serum levels of Alp, Oc, and Ck. The Alp level was significantly higher in all OVX groups compared to that in intact rats in both experiments (all OVX groups 79 ± 5 U/L, intact 59 ± 3 U/L). The level of Oc and Ck did not differ (*P* > 0.05) among the groups (19 ± 1 and 10,111 ± 1,160 U/L on average, respectively).

### Bone Healing Analyses

Biomechanical analysis did not reveal differences between the treatment groups in either of the experiments. Stiffness was 45 ± 5 and 69 ± 17 N/mm in intact rats and 49 ± 3 and 47 ± 6 N/mm in all OVX groups on average in experiments 1 and 2, respectively. The yield load averaged 26 ± 3 and 34 ± 8 N in the OVX groups; in sham rats the yield load was measured at 25 ± 3 and 32 ± 7 N in experiments 1 and 2, respectively.

Micro-CT analyses showed that vibration did not affect the total BMD in OVX rats in experiment 1 (Table 1). BMD was lower in the 35-, 70-, and 90-Hz groups than in the intact group. BV/TV increased in the 35- and 50-Hz groups to the same level as that in intact rats. In experiment 2, there were no differences in total BMD among the groups (Table 2). BV/TV was significantly lower in all OVX groups irrespective of the vibration treatments. The callus and cortical densities did not differ between the groups (*P* > 0.05) in both experiments (data not shown).

**Table 1:** Vertical WBV: micro-CT and microradiographic analyses of the tibia at the osteotomy site divided into ventral, dorsal, and endosteal regions in intact and OVX rats exposed to the different vibrations (at least nine replications/treatment group, experiment 1)

	Intact		OVX		35 Hz		50 Hz		70 Hz		90 Hz	
	Mean	SEM	Mean	SEM	Mean	SEM	Mean	SEM	Mean	SEM	Mean	SEM
Micro-CT												
Total denisty (g/cm^3^)	1.50^a^	0.04	1.45^ab^	0.06	1.37^b^	0.02	1.45^ab^	0.04	1.36^b^	0.02	1.38^b^	0.02
BV/TV (%)	59^a^	4	47^b^	2	52^ab^	2	55^ac^	3	50^bc^	2	51^bc^	2
Microradiogpraphy												
Ventral												
Cortical width (mm)	0.47	0.02	0.51	0.03	0.50	0.02	0.51	0.02	0.51	0.02	0.53	0.02
Cortical density (%)	100^a^	0.02	95^b^	0.6	97^c^	0.3	98^c^	0.3	98^c^	0.2	97^c^	0.3
Callus width (mm)	0.45^a^	0.03	0.57^bc^	0.04	0.52^ab^	0.04	0.56^bc^	0.03	0.64^c^	0.04	0.62^bc^	0.03
Callus density (%)	86^a^	1.6	72^b^	1.8	66^b^	2.4	65^b^	3.3	72^b^	2.8	69^b^	2.8
Dorsal												
Cortical width (mm)	0.77	0.04	0.76	0.04	0.68	0.03	0.74	0.03	0.77	0.03	0.80	0.05
Cortical density (%)	100^a^	0.01	97^b^	0.4	98^cd^	0.4	99^d^	0.2	98^d^	0.2	97^bc^	0.4
Callus width (mm)	0.71^a^	0.04	0.74^a^	0.03	0.76^a^	0.05	0.89^bc^	0.05	0.94^c^	0.05	0.79^ab^	0.04
Callus density (%)	92^a^	1.3	71^b^	2.1	77^c^	1.8	80^c^	2.1	81^c^	1.6	76^bc^	1.9
Endosteal												
Callus density (%)	90^a^	1.6	65^b^	1.5	67^bc^	2.6	71 ^cd^	2.7	82^e^	1.3	73^d^	1.6
Trabecular density (%)	13^a^	2.1	2^b^	0.4	3^b^	0.7	4^b^	0.7	4^b^	0.4	3^b^	1.0
Trabecular width (μm)	4.5^a^	0.2	2.5^b^	0.2	2.5^b^	0.3	2.2^b^	0.2	2.3^b^	0.1	2.5^b^	0.2

^a–e^Between treatment groups, means with different superscripts differ significantly (*P* < 0.05, Scheffé test)

**Table 2:** Horizontal WBV: micro-CT and microradiographic analyses of the tibia at the osteotomy site divided into ventral, dorsal, and endosteal regions in intact and OVX rats exposed to the different vibrations (at least 11 replications/treatment group, experiment 2)

	Intact		OVX		30 Hz		50 Hz		70 Hz		90 Hz	
	Mean	SEM	Mean	SEM	Mean	SEM	Mean	SEM	Mean	SEM	Mean	SEM
Micro-CT												
Total denisty (g/cm^3^)	1.37	0.04	1.27	0.02	1.31	0.03	1.32	0.02	1.29	0.02	1.30	0.04
BV/TV (%)	42^a^	3	34^b^	2	34^b^	2	32^b^	2	32^b^	3	29^b^	1
Microradiogpraphy												
Ventral												
Cortical width (mm)	0.47^ab^	0.02	0.49^a^	0.02	0.43^bc^	0.02	0.41^c^	0.02	0.43^bc^	0.02	0.45^ac^	0.02
Cortical density (%)	100	0.04	100	0.14	99	0.17	99	0.25	100	0.11	100	0.12
Callus width (mm)	0.50	0.04	0.48	0.03	0.51	0.03	0.51	0.08	0.47	0.04	0.40	0.02
Callus density (%)	79^a^	2	68^b^	2	62^b^	3	66^b^	3	66^b^	3	66^b^	3
Dorsal												
Cortical width (mm)	0.51	0.05	0.55	0.03	0.62	0.04	0.59	0.02	0.56	0.04	0.64	0.03
Cortical density (%)	100	0.09	100	0.10	99	0.24	99	0.36	100	0.14	100	0.08
Callus width (mm)	1.02	0.14	0.79	0.08	0.90	0.07	0.86	0.05	0.78	0.05	0.95	0.07
Callus density (%)	83^a^	2	76^ab^	3	66^c^	3	73^b^	2	74^b^	3	76^ab^	2
Endosteal												
Callus density (%)	82	3	67	3	62	3	62	3	66	3	71	3
Trabecular density (%)	20^a^	3	10^b^	3	10^b^	2	9^b^	2	8^b^	1	8^b^	2
Trabecular width (μm)	5^a^	0.3	4^b^	0.3	3^b^	0.2	3^b^	0.2	4^b^	0.3	3^b^	0.2

^a–c^Between treatment groups, means with different superscripts differ significantly (*P* < 0.05, Scheffé test)

In experiment 1, analyses of microradiographs of the histological sections revealed enhanced cortical density in the OVX rats exposed to vibrations below 90 Hz compared to the OVX group (Table 1). In the latter group, cortical density was significantly lower than in intact rats. Cortical width was affected by neither ovariectomy nor vibration. Endosteal callus density was improved after vibration treatments of 50, 70, and 90 Hz; and at the dorsal aspect vibrations below 90 Hz enhanced callus density. Callus width at the dorsal aspect of the tibia increased following treatments with 50- and 70-Hz vibrations. In experiment 2, the reduced cortical width at the ventral aspect and the callus density at the dorsal aspect were measured in rats treated with vibrations at frequencies below 90 Hz (Table 2). The vibration at 90 Hz did not change these parameters. Callus width and cortical density did not change between the groups.

In both experiments, trabecular density and width were not affected by the vibration treatments. Ovariectomy significantly diminished callus and trabecular parameters of the metaphyseal tibia.

**Fig. 2:**
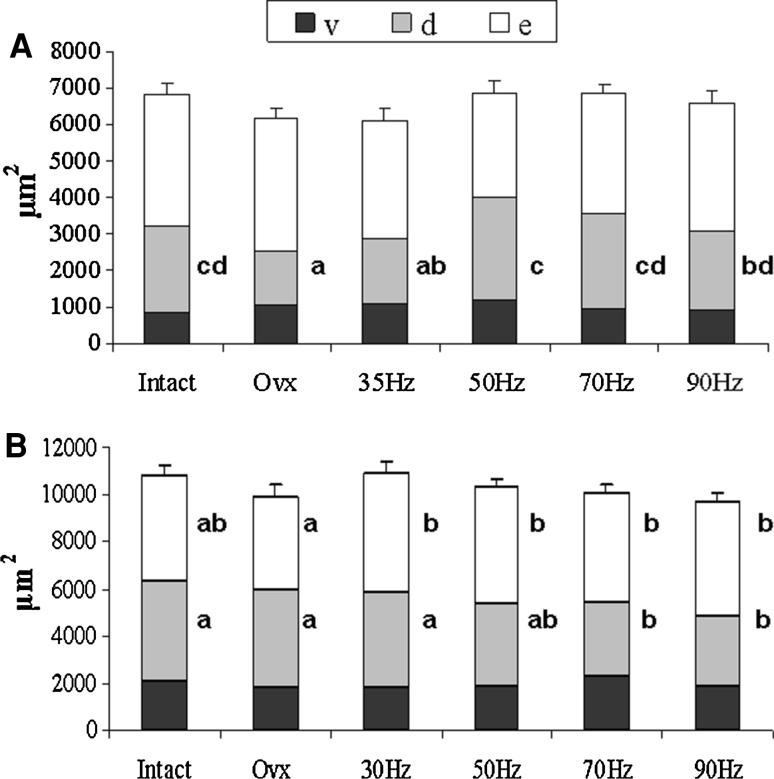
Total callus area (μm^2^) measured in fluorescence-labeled sections of the tibia divided into ventral (*v*), dorsal (*d*), and endosteal (*e*) regions in intact and OVX rats exposed to the different vibrations (30–90 Hz). Means with different letters differ significantly between the treatment groups, at the dorsal or endosteal region (*P* < 0.05, Scheffé test). **a** Vertical WBV (experiment 1), **b** horizontal WBV (experiment 2)

Fluorescence analysis of the labeled callus revealed that in experiment 1 the first osseous bridging of the osteotomy gap occurred in intact rats at day 23, in OVX rats at day 26, in 35-Hz rats at day 22, in 50-Hz rats at day 23, and in 70- and 90-Hz rats at day 26. In experiment 2 the first osseous bridging of the osteotomy gap occurred in intact, 30-, 70-, and 90-Hz rats on days 18, 20, 21, and 22, respectively. In the OVX and 50-Hz groups, osseous bridging occurred on day 23 after osteotomy. Bridging of osteotomized bone ends was observed in all rats. The total callus area did not differ between the groups in either experiment (Fig. 2a, b). In experiment 1 the callus area was enlarged after vibrations of above 35 Hz in OVX rats (Fig. 2a). In experiment 2 the callus area was reduced in the 70- and 90-Hz groups at the dorsal aspect, whereas the endosteal callus area was increased after all vibration treatments compared to the nonvibrated OVX rats (Fig. 2b). In general, the rate of callus formation was the highest at the third week of the healing period (data not shown).

### Analyses of Gene Expression at the Osteotomy Site

**Fig. 3:**
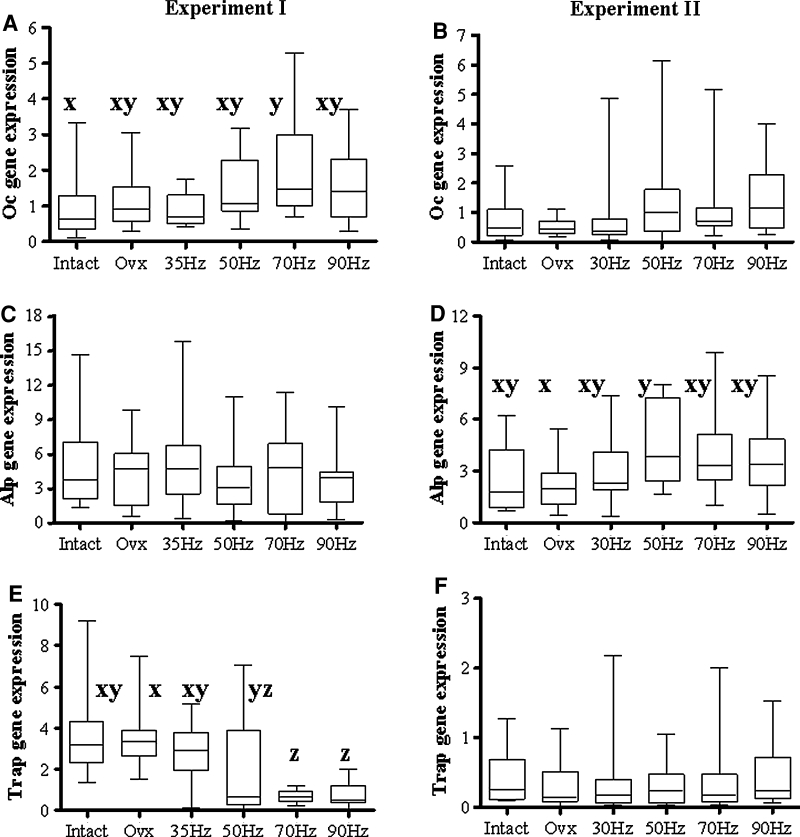
*Box plot* illustrating the relative mRNA expression level of **a**, **b** Oc, **c**, **d** Alp, and **e**, **f** Trap genes in OVX rats, either untreated or exposed to the vertical (*first column*, experiment 1) or horizontal (*second column*, experiment 2) vibration treatment, and intact rats, calculated using the 2^−ΔΔCT^ method. Each treatment was conducted with at least 10 replications. Medians with different letters differ significantly (*P* < 0.05, Dunn test)

In experiment 1 the Oc gene was upregulated in all OVX groups, reaching a significant level in the 70-Hz group compared to the intact group (Fig. 3a). Expression of the Trap gene was lower in the groups that were exposed to vibrations above 35 Hz than in nonvibrated OVX rats (Fig. 3b). In experiment 2 the Alp gene was upregulated in the 50-Hz group compared to the OVX group (Fig. 3d). The differences in expression of the Opg, Rankl, and Igf-1 genes were not significant between the treatment groups in either experiment (data not shown).

### Muscle Analyses

The weights of the MG and MS did not differ significantly among the groups in either experiment. The MG weight was 1.99 g (SEM = 0.9) on average, and the MS weight averaged 0.13 ± 0.01 g. In experiment 1, the MG/BW ratio increased in the 50-Hz group to the level observed in intact rats, whereas in the other groups the MG/BW ratio was lower (Table 3). In experiment 2 differences in the MG/BW ratio were not detected (Table 4).

**Table 3:** Vertical WBV: muscle weight to BW ratio, cross-sectional area (CSA) of the glycolytic (G) and oxidative (O) fibers, and activity of LDH, CS, and complex I in the gastrocnemius (MG) and soleus (MS) muscles of intact and OVX rats exposed to the different vibrations (at least nine replications per treatment group, experiment 1)

	Intact		OVX		35 Hz		50 Hz		70 Hz		90 Hz	
	Mean	SEM	Mean	SEM	Mean	SEM	Mean	SEM	Mean	SEM	Mean	SEM
Muscle weight to BW (mg/g)												
MG/BW	6.9^a^	0.1	6.2^bc^	0.1	6.3^bc^	0.2	6.5^ab^	0.2	6.3^bc^	0.1	5.9^c^	0.2
MS/BW	0.47	0.03	0.40	0.01	0.44	0.02	0.45	0.02	0.42	0.01	0.42	0.01
Histology												
MG												
CSA, G (μm^2^)	4,723	152	4,468	263	4,838	178	4,926	127	4,280	214	4,600	222
CSA, O (μm^2^)	1,673	106	1,616	102	1,803	102	1,754	77	1,613	116	1,676	88
Capillaries/fiber	0.78	0.17	0.82	0.25	0.86	0.23	1.01	0.18	0.87	0.20	0.95	0.28
MS												
CSA, O (μm^2^)	4,687	109	4,939	244	5,517	240	4,329	218	5,032	485	5,242	282
Capillaries/fiber	0.90	0.19	1.05	0.21	1.09	0.29	0.98	0.31	1.17	0.26	1.14	0.22
Enzymes												
MG												
LDH (U/mg)	2.0	0.1	2.0	0.2	2.0	0.1	2.2	0.2	2.0	0.2	1.9	0.1
CS (U/g)	55	5	54	3	56	6	49	4	55	4	67	7
Complex I (U/g)	3.8	0.7	4.0	0.7	4.2	0.8	2.3	0.4	2.6	0.6	3.9	0.7
MS												
LDH (U/mg)	0.4	0.01	0.4	0.04	0.4	0.03	0.4	0.02	0.4	0.03	0.4	0.05
CS (U/g)	29^a^	4	31^ab^	3	41^bc^	5	45 ^cd^	5	47 ^cd^	4	56^d^	2
Complex I (U/g)	8.6	1.3	7.9	0.8	7.7	0.8	6.4	0.4	7.8	0.9	7.6	1.2

^a–d^Between treatment groups, means with different superscripts differ significantly (*P* < 0.05, Scheffé test)

**Table 4:** Horizontal WBV: muscle weight to BW ratio, cross-sectional area (CSA) of the glycolytic (G) and oxidative (O) fibers, and activity of LDH, CS, and complex I in the gastrocnemius (MG) and soleus (MS) muscles of intact and OVX rats exposed to the different vibrations (at least 11 replications/treatment group, experiment 2)

	Intact		OVX		35 Hz		50 Hz		70 Hz		90 Hz	
	Mean	SEM	Mean	SEM	Mean	SEM	Mean	SEM	Mean	SEM	Mean	SEM
Muscle to BW ratio (mg/g)												
MG/BW	6.5	0.2	6.1	0.3	6.3	0.2	6.3	0.2	6.5	0.1	6.3	0.2
MS/BW	0.41	0.01	0.36	0.01	0.40	0.02	0.39	0.01	0.37	0.02	0.39	0.04
Histology												
MG												
CSA, G (μm^2^)	5,771	185	5,975	177	5,728	241	5,673	194	5,578	187	5,811	238
CSA, O (μm^2^)	2,502^a^	110	2,456^ab^	84	2,192^c^	69	2,442^ad^	102	2,041^c^	68	2,228^bcd^	88
Capillaries/fiber	1.59	0.06	1.70	0.11	1.77	0.09	1.70	0.08	1.82	0.06	1.44	0.17
MS												
CSA, O (μm^2^)	4,097	333	5,052	455	4,284	332	4,711	247	4,902	467	4,788	467
Capillaries/fiber	1.70^a^	0.08	1.96^ab^	0.14	2.02^bc^	0.05	2.02^bc^	0.10	2.26^c^	0.09	2.54^d^	0.10
Enzymes												
MG												
LDH (U/mg)	3.8	0.3	3.2	0.2	3.4	0.3	3.2	0.2	3.2	0.2	3.3	0.2
CS (U/g)	60	8	81	7	76	8	79	7	83	8	80	8
Complex I (U/g)	4.8	1.2	6.7	1.3	5.5	0.8	7.4	1.5	6.1	1.0	6.1	1.3
MS												
LDH (U/mg)	0.4	0.03	0.4	0.04	0.4	0.05	0.5	0.09	0.4	0.06	0.4	0.05
CS (U/g)	87	12	89	14	86	11	103	15	88	12	90	7
Complex I (U/g)	13.7	2.7	10.8	1.8	10.0	1.4	10.8	2.8	9.3	1.7	10.2	1.1

^a–d^Between treatment groups, means with different superscripts differ significantly (*P* < 0.05, Scheffé test)

In experiment 1 neither muscle fiber size nor capillary density differed significantly between the treatment groups in either of the muscles that were studied (Table 3). In experiment 2 the CSA of the oxidative fibers decreased in the MG from rats in the 35- and 70-Hz groups compared to that from rats in the OVX and intact groups (Table 4). The size of the G fibers did not change among the groups. Capillary density was significantly enhanced in the MS of rats from the 70- and 90-Hz groups compared to the OVX and intact groups (Table 4).

The activity of CS increased gradually with increasing frequencies of vertical vibration in the MS, reaching a significant level in the 35-Hz group compared to intact rats (Table 3). Compared to the OVX group, vibration exerted a significant effect at 50 Hz and higher. In the MG, CS activity did not change between the groups. The LDH and complex I activities did not differ significantly between the groups in either the MG or the MS. In experiment 2, there were no differences detected in the activities of the enzymes (Table 4).

## Discussion

Several reviews on WBV application for the improvement of the musculoskeletal system and bone healing have revealed the need for systematic studies to investigate variations in the vibration characteristics and the optimal dose–response relationship to determine the underlying mechanisms of muscle and bone adaptations, efficacy, and clinical relevance [3, 9, 24, 25]. The present study was conducted to compare for the first time the effectiveness of either horizontal or vertical WBV over a wide range of frequencies for promoting bone healing and muscle function in ovariectomy-induced osteopenic rats. Ovariectomy was confirmed by atrophied uterine horns. Increased BW and food intake in the OVX rats have been previously reported after ovariectomy [13]. Short exposure to vertical and horizontal vibrations exerted no effect on the BW or food intake of rats.

### Bone Healing

In general, vertical vibration treatments improved cortical and callus densities to some extent and enlarged the periosteal callus area and width. Horizontal vibrations did not ameliorate the impaired cortical and callus parameters in OVX rats. Moreover, horizontal vibrations reduced the cortical width at the ventral aspect and callus area and dorsally reduced the density. In these vibrated rats, the better stabilization of the osteotomy gap was most likely achieved due to the increased endosteal callus area. Although a biomechanically stable fixation was used, different vibrations could evoke movements at the fracture site, which may have differing effects on bone healing. Previously, it was reported that axial micromovements of fractured bone ends, such as those that occur with weight bearing, compression force, or bending movements, are favorable for fracture healing [26, 27], whereas shearing movements are injurious [28]. We generated movements in either the vertical or the horizontal plane. However, the force was applied indirectly to the fracture site and transmitted via the surrounding tissues and T-plate. The effect of horizontal force on fracture healing was ambiguous. Additional studies are under way to clarify this issue.

Rat limbs are naturally exposed to forces in a vertical direction during movement or jumping and weight bearing, whereas horizontal stimuli seldom occur in nature. Some authors have demonstrated increased callus area and density at the fracture site after mechanical stimulation, such as 2D (vertical and horizontal) vibration of 25 Hz for 20 or 60 min/day in rabbit fibula [29] and vertical vibration of 35 Hz for 20 min/day in rat femora [30]. Wolf et al. [31] reported no effect of daily WBV (20 Hz, 5 min) on healing of ovine metatarsus. These authors investigated skeletally mature and healthy animals. In OVX rats, a WBV of 35 Hz (20 min, 5 days/week) promoted callus formation, mineralization, and remodeling [32, 33]. In our previous study, we showed that a vertical vibration regime (90 Hz, 15 min twice a day) improved musculoskeletal tissue but that it was not favorable for bone healing in OVX rats [10].

Micro-CT analysis has been shown to have many advantages and is accepted as an alternative to histological analysis, which is considered the gold-standard method of examination of bone specimens [34]. In this study, histological analyses permitted a more detailed assessment of the callus parameters and bone healing than micro-CT. Further development of computer software for micro-CT is under way to better assess cortical and callus tissues at the osteotomy site. The decreased total density determined by micro-CT in the vibrated groups could be explained by the enlarged callus volume in these groups, which was less compact than the cortical bone. Density was calculated over the entire volume.

The biomechanical test did not reveal differences between the treatment groups in either experiment. As previously stated, the callus biomechanical properties do not correlate with histomorphological parameters [10, 12]. Callus quality may depend on a noncalcified callus, which was not assessed histologically. Analysis of fluorochrome-labeled sections showed that the vibration treatments did not impede osseous bridging of the osteotomy gap, which occurred during the third and fourth weeks after osteotomy. Moreover, the vibration treatments of 35 and 50 Hz (vertical) accelerated the delayed osteotomy bridging in OVX rats.

Based on our investigation of mRNA expression of bone-related genes in the callus, different responses were observed after vertical and horizontal vibration treatments. Vertical vibration caused enhanced expression of the osteoblast gene Oc (70 Hz) and diminished expression of the osteoclast gene Trap (>35 Hz). This indicates that the callus and bone tissues at the osteotomy site were sensitive to the vertical vibrations, and their response suggested enhanced bone synthesis in these rats. Similar upregulation of the Oc gene was reported in the callus of OVX rats after 90-Hz vibrations [10]. Differences in serum Oc levels were not detected. The gene-expression level does not directly correspond to the level of protein synthesis [35]. In rats exposed to horizontal vibrations, enhanced mRNA expression of Alp was detected. This may be a sign at the molecular level that the state of impaired bone turnover in OVX rats could be worsened after horizontal vibration. The elevated serum levels of Alp detected in both experiments can be explained by the enhanced rate of bone turnover in the OVX rats [10, 11].

In the present study, we reported that the most favorable frequencies for vertical vibration were 35 and 50 Hz. The effects of 70- and 90-Hz vibrations on bone healing were not clearly defined. Some callus and cortical parameters were improved, and the expression of bone genes could favor bone synthesis; however, bridging of the osteotomy gap occurred at the same time as in the nonvibrated OVX rats. The 90-Hz vibration applied for 15 min twice a day was shown to impair bone healing [10]. In contrast, for nonosteotomized bone, vertical vibration at 90 Hz caused a stronger anabolic response than vibration at 45 Hz [36]. This should be taken into consideration when WBV treatments are applied to ameliorate osteoporotic bone parameters and from the perspective of bone healing.

### Muscle

Similarly to the bone healing studies, muscle tissue responded to the vibration treatments differently. Reduction of the muscle to BW ratio was observed after ovariectomy in the MG, whereas vertical WBV of 50 Hz returned the muscle to BW ratio to the level measured in intact rats (experiment 1). These changes occurred independently of fiber size. In the muscle of mature female rodents, the CSAs of all types of fibers were reported to be unaffected by estrogen status [37, 38]. Altered metabolism in the OVX rats may be responsible for the increase in adipose tissue and BW [39]. This may lead to the reduced muscle to BW ratio, whereas vertical vibration demonstrated a favorable effect on this parameter in the OVX rats.

Whereas the vertical vibrations did not affect the muscle structure, horizontal forces reduced the CSA of oxidative fibers, reaching a significant level at 35, 70, and 90 Hz in MG. These changes in fiber size did not correspond to the unchanged muscle weight and BW. The enhanced capillary density may be explained by the mechanical stimulus, which may have increased the blood flow and capillary shear stress, promoting angiogenesis in the muscle [40]. On the other hand, an increase in capillary density was reported to be related to the decreased fiber size [20].

In general, the metabolic enzymes studied here were not changed after the vibration treatments. The exception was CS, which increased after vertical vibrations in the MS. This may be explained by the enhanced oxidative function of the MS. The soleus muscle consists of predominantly oxidative fibers, which have the largest mean volume fraction of mitochondria among muscle fibers [18]. CS is an enzyme that is involved in the first step of the citric acid cycle. This enzyme catalyzes the condensation of acetyl-coenzyme A and oxaloacetate to citrate accompanied by the liberation of acetyl-coenzyme A. CS activity is commonly used to assess metabolic capacity, including the oxidative and respiratory capacities of muscle, and the mitochondrial volume density [18]. The level of CS activity after exercise training has been reported to change from 0 to 100 % [41]. In this context, the timing of muscle sampling relative to the last exercise session is critical when measuring CS [41]. In both experiments of the present study, the last exposure to the vibration treatments was performed 1 day before sample collection. Several studies on rat skeletal muscle have reported unaltered or even decreased CS levels immediately after acute exercise [42]. Savard et al. [43] reported a 30 % increase in CS when rat muscle was harvested 24 h after acute exercise.

Metabolic adaptations to endurance exercises are believed to increase the potential of the citric acid cycle, to enhance lipid utilization, and to decrease glycolysis [44]. In the present study, neither the glycolytic enzyme (LDH) nor the oxidative phosphorylation enzyme (complex I) differed after vibration treatments. These findings indicate that the vibration treatments did not produce a sudden change in the environment of rats as a result of a sharp increase in the level of physical activity [45], and the adaptive stimulus did not alter the functional cell properties. Furthermore, the vibration treatments did not change the serum levels of Ck, an indicator of muscle injury and other muscle diseases.

## Conclusion

One of the proposed mechanisms by which WBV exerts its effects on bone tissue is the activation of muscle, which mechanically loads the bone [2, 8]. Another hypothesis is that WBV signals become amplified within the bone tissue by stress-generated fluid flow, thereby activating bone cells, which act as mechanosensors [2, 8]. In the present study, although the osteotomized bone was fixed by a T-plate, the vibration stimuli may have directly affected bone healing by evoking the movements of the osteotomized bone ends. Vertical WBV appeared to be favorable both for bone healing and for muscle tissue. The preferable frequencies were 35 and 50 Hz, whereas 70 and 90 Hz showed less effect. Horizontal WBV was found to exert no positive effect, and it affected some of the bone and muscle parameters unfavorably. These findings indicate that the type of vibration, which is often not reported in the literature [24], is an important parameter. The vibration parameters applied at rehabilitation institutes using vibration plates are largely determined by the manufacturer’s recommendations. The periodicity is often selected based on common routines for strength training [1]. However, WBV protocols should be designed specifically for the treatment (improvement of osteoporotic bone structure, built-up or conservation of skeletal muscles after trauma) and for the patients, who may differ in age, hormonal status [24], bone and muscle state, fracture type, etc. Until the underlying mechanisms of the effects of WBV on different physiological systems have been elucidated, precautions have to be taken when applying WBV as a therapy for postmenopausal women and as a possible means for improvement of bone healing.
